# Circulating cell-free DNA as a prognostic and predictive biomarker in non-small cell lung cancer

**DOI:** 10.18632/oncotarget.10069

**Published:** 2016-06-15

**Authors:** Bo Ai, Huiquan Liu, Yu Huang, Ping Peng

**Affiliations:** ^1^ Department of Thoracic Surgery, Tongji Hospital, Tongji Medical College, Huazhong University of Science and Technology, Hubei, Wuhan 430030, People's Republic of China; ^2^ Department of Oncology, Tongji Hospital, Tongji Medical College, Huazhong University of Science and Technology, Hubei, Wuhan 430030, People's Republic of China

**Keywords:** circulating cell-free DNA, non-small cell lung cancer, prognosis, biomarker, meta-analysis

## Abstract

Circulating cell-free DNA (cfDNA), which can be obtained from plasma or serum by non-invasive procedures, has showed great potential to predict treatment response and survival for cancer patients. Several studies have assessed the prognostic and predictive value of cfDNA in non-small cell lung cancer (NSCLC). However, these studies were often small and reported varying results. To address this issue, a meta-analysis was carried out. A total of 22 studies involving 2518 patients were subjected to the final analysis. Our results indicated that NSCLC patients with higher cfDNA concentration had shorter median progression-free survival (PFS) and overall survival (OS) time. In addition, high levels of cfDNA were significantly associated with poor PFS (hazard ratio or HR, 1.32; 95% CI, 1.02-1.71) and OS (HR, 1.64; 95% CI, 1.26-2.15). With respect to tumor specific mutations, we failed to reveal significant differences for PFS (HR, 1.30; 95% CI, 0.66-2.56) and OS (HR, 1.05; 95% CI, 0.49-2.25) when NSCLC patients were grouped according to *KRAS* genotype detected in cfDNA. However, NSCLC patients which harbored *EGFR* activating mutation in cfDNA had a greater chance of response to EGFR-TKIs (odds ratio or OR, 1.96; 95% CI, 1.59-2.42). No significant publication bias was detected in this study. In conclusion, cfDNA could act as a prognostic and predictive biomarker for patients with NSCLC.

## INTRODUCTION

Lung cancer is the most commonly diagnosed cancer as well as the leading cause of cancer-related deaths in the world [1]. Non-small cell lung cancer (NSCLC) accounts for approximately 80% cases of lung cancer [2]. Most NSCLC patients are diagnosed with advanced or distant stages and they are ineligible for curative surgery and often suffer a poor survival. Identifying biomarkers related to treatment response and prognosis may be helpful to improve the clinical outcome of patients with NSCLC.

Circulating cell-free DNA (cfDNA), which can be isolated from the plasma or serum by non-invasive procedures, has been proposed as an attractive biomarker to estimate treatment response, detect drug resistance and predict clinical outcome for cancer patients [3–7]. It has been experimentally evidenced that tumor cells can release genomic DNA into the blood and circulating DNA can reflect the tumor burden and tumor biologic characteristics [6, 8]. A series of studies have shown that NSCLC patients have higher levels of cfDNA in the blood compared with healthy controls or patients with benign diseases [9–11]. The quantitative assay of cfDNA may be a screening tool for NSCLC. It has been shown that the diagnostic accuracy of quantitative analysis of cfDNA is not lower than conventional serum biomarkers for lung cancer screening [12]. Furthermore, cancer-associated genetic alterations, such as point mutations, deletions, and copy number variations, can be detected in cfDNA [13]. In NSCLC, many studies have investigated the diagnostic accuracy of cfDNA for detecting epithermal growth factor receptor (*EGFR*) mutation [14–16]. Two recent meta-analyses demonstrated that cfDNA was a highly specific and effective biomarker to measure *EGFR* mutation status in NSCLC [17, 18]. These evidences suggested that genotype in cfDNA could be a promising tumor biomarker for NSCLC.

A large number of studies had investigated the predictive or prognostic value of cfDNA concentration in NSCLC patients in recent years [19–22] (see Table 1 for references). However, these studies were often small and reported varying results. Some of them showed that a higher cfDNA concentration was associated with poorer survival in NSCLC patients [19, 20], whereas other studies failed to demonstrate such correlation [21, 22]. On the other hand, several studies had analyzed the association between genotype detected in cfDNA with treatment response or survival in NSCLC [23–26]. Some of them suggested that tumor specific mutations such as *KRAS* or *EGFR* presented in cfDNA might be useful prognostic and predictive biomarkers for NSCLC [23, 24]. However, some others indicated that such gene mutations in cfDNA had no predictive or prognostic value [25, 26].

**Table 1:** Characteristics of studies included in this meta-analysis

First author	Country	No.	Clinical stage	Therapeutic regimen	cfDNA assessments	cfDNA analysis	Clinical factors
Catarino(2012)^9^	Portugal	104	I-IV	chemotherapy	qPCR(hTERT)	quantification(H/L)	OS
Tissot(2015)^19^	France	218	III-IV	chemotherapy	PicoGreen dsDNA Kit	quantification(H/L)	PFS, OS
Nygaard(2014)^20^	Denmark	58	III-IV	chemotherapy	ARMS-qPCR	quantification(H/L)	PFS, OS
						*KRAS* mutation(+/−)	PFS, OS
Bortolin(2015)^21^	Italy	22	I	stereotactic body radiotherapy	qPCR(hTERT)	quantification(H/L)	PFS, OS
Li(2016)^22^	America	101	III-IV	chemotherapy	qPCR(β-Actin)	quantification(H/L)	PFS, OS
Wang(2014)^27^	China	134	III-IV	EGFR-TKI	ARMS/Scorpion assay	quantification(H/L)	PFS, OS
						*EGFR* mutation(+/−)	PFS, OS
Vinayanuwattikun (2013)^28^	Thailand	58	III-IV	chemotherapy	qPCR(GAPDH)	quantification(H/L)	OS
Sirera(2011)^29^	Spain	446	III-IV	chemotherapy	qPCR(hTERT)	quantification(H/L)	PFS, OS
Lee(2011)^30^	Korea	134	III-IV	EGFR-TKI or chemotherapy	qPCR(β-Actin)	quantification(H/L)	PFS, OS
Ludovini(2008)^31^	Italy	76	I-III	surgery+ chemotherapy	qPCR(hTERT)	quantification(H/L)	PFS, OS
Camps(2006)^32^	Spain	99	III-IV	chemotherapy	qPCR(hTERT)	quantification(H/L)	PFS, OS
Gautschi(2004)^33^	Switzerland	185	I-IV	chemotherapy	Fluorogenic qPCR	quantification(H/L)	OS
Nygaard(2013)^23^	Denmark	246	II-IV	chemotherapy	ARMS-qPCR	*KRAS* mutation(+/−)	PFS, OS
Camps(2005)^25^	Spain	67	III-IV	chemotherapy	RFLP-PCR	*KRAS* mutation(+/−)	PFS, OS
Gautschi(2007)^34^	Switzerland	175	I-IV	surgery+ chemotherapy	RFLP-PCR	*KRAS* mutation(+/−)	OS
Bai(2009)^16^	China	102	III-IV	EGFR-TKI	DHPLC	*EGFR* mutation(+/−)	ORR
Kimura(2007)^24^	Japan	42	III-IV	EGFR-TKI	DNA sequencing	*EGFR* mutation(+/−)	ORR
Douillard(2014)^26^	Multicenter	102	III-IV	EGFR-TKI	EGFR RGQ PCR kit	*EGFR* mutation(+/−)	ORR
He(2009)^35^	China	45	I-IV	EGFR-TKI	Mutant-enriched PCR	*EGFR* mutation(+/−)	ORR
Kimura(2006)^36^	Japan	27	III-IV	EGFR-TKI	DNA sequencing	*EGFR* mutation(+/−)	ORR
Kim(2013)^37^	Korea	22	III-IV	EGFR-TKI	PNA-LNA PCR	*EGFR* mutation(+/−)	ORR
Li(2014)^38^	China	55	III-IV	EGFR-TKI	ARMS-qPCR	*EGFR* mutation(+/−)	ORR

Abbreviation: No., number; cfDNA, circulating cell-free DNA; qPCR, quantitative polymerase chain reaction; hTERT, human telomerase reverse transcriptase; ARMS, amplification refractory mutation system; GAPDH, glyceraldehyde-phosphate dehydrogenase; RFLP, restricted fragment length polymorphisms; DHPLC, denaturing high-performance liquid chromatography; PNA-LNA, peptide nucleic acid-locked nucleic acid; EGFR-TKI, epidermal growth factor receptor-tyrosine kinase inhibitor; H/L, high/low; +/−, mutation/wide-type; OS, overall survival; PFS, progression-free survival; ORR, objective response rate.

As the existing studies are conflicting in their results, it is still difficult to determine the predictive and prognostic role of cfDNA in patients with NSCLC. Therefore, a meta-analysis aimed to address this issue was carried out.

## RESULTS

### Search results

**Figure 1:**
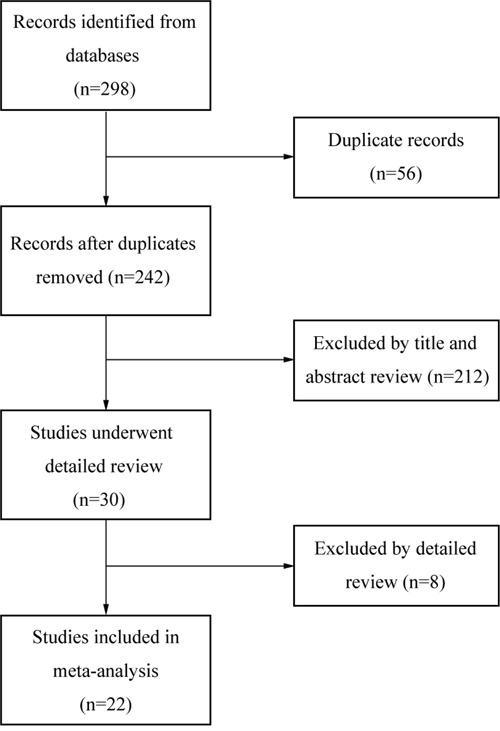
Flow diagram of study selection

Figure 1 illustrated the process of study selection. 298 studies were initially found by our search strategy. 30 articles were reviewed in detail after the article titles and abstracts were checked [9, 16, 19–46]. Eight studies were excluded from the meta-analysis [39–46], leaving 22 studies that fulfilled the eligibility criteria [9, 16, 19–38] (Table 1). Among the 8 excluded studies, 7 did not provide sufficient data for extracting odds ratio (OR) or hazard ratio (HR) [39–45], and other 1 study was excluded because the same cohort of patients was used in other selected study [46]. The total number of patients included in this study was 2518, ranging from 22 [21, 37] to 446 [29] cases per study. 12 studies evaluated the prognostic role of cfDNA concentration in NSCLC [9, 19–22, 27–33]. 4 studies reported the prognostic role of *KRAS* genotype detected in cfDNA for NSCLC [20, 23, 25, 34]]. Another 7 studies dealt with the predictive role of *EGFR* genotype presented in cfDNA for NSCLC patients who were treated with tyrosine kinase inhibitors of EGFR (EGFR-TKIs) [16, 24, 26, 35–38].

### Impact of cfDNA concentration on the survival of NSCLC

**Figure 2:**
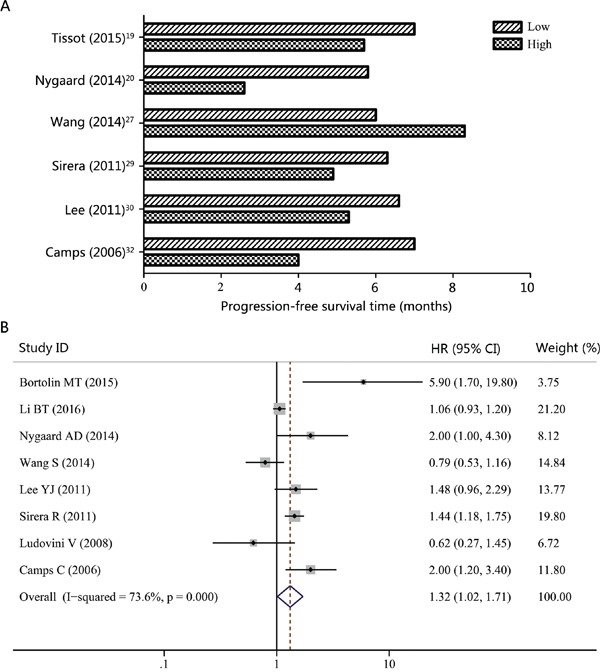
Progression-free survival (PFS) according to cfDNA concentration in NSCLC patients **A.** Median PFS time according to cfDNA concentration. **B.** Forest plot of hazard ratio (HR) for the impact of cfDNA concentration on PFS.

**Figure 3:**
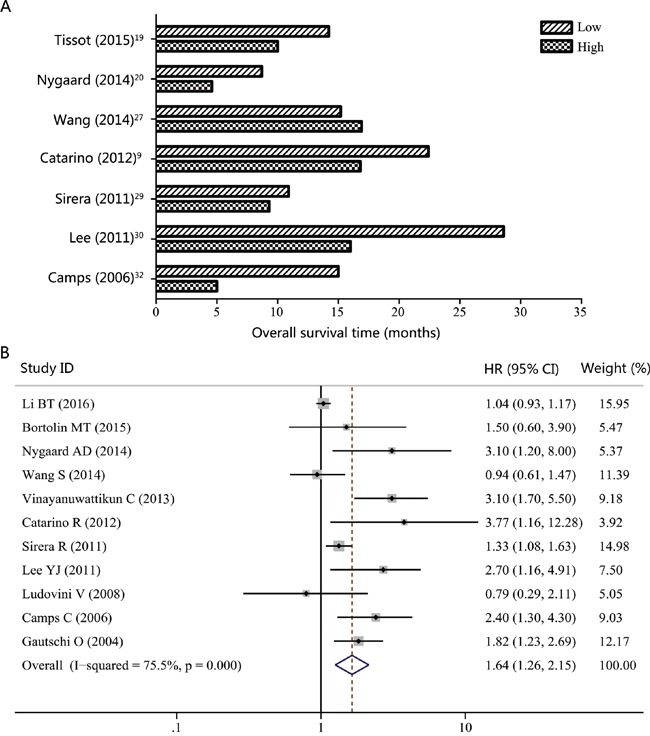
Overall survival (OS) according to cfDNA concentration in NSCLC patients **A.** Median OS time according to cfDNA concentration. **B.** Forest plot of hazard ratio (HR) for the impact of cfDNA concentration on OS.

Six studies reported the median progression-free survival (PFS) time in NSCLC patients according to different cfDNA concentrations (high or low) [19, 20, 27, 29, 30, 32]. As showed in Figure 2A, patients with high levels of cfDNA usually had shorter PFS time than those with low cfDNA concentrations. In addition, the pooled HR for PFS was 1.32 (95% CI, 1.02-1.71; *P* = 0.038), suggesting that high cfDNA concentration was a good predictor of poor PFS (Figure 2B). For overall survival (OS), 6 of 7 studies reported shorter median OS times in NSCLC patients with higher cfDNA concentration (Figure 3A). Similar to the results of PFS, higher levels of cfDNA indicated lower overall survival rates with a pooled HR of 1.64 (95% CI, 1.26-2.15; *P* = 0.000) (Figure 3B). However, high heterogeneities were presented in these analyses (*I^2^* = 73.6%; *P =* 0.000 for PFS; *I^2^* = 75.5%; *P =* 0.000 for OS).

**Figure 4:**
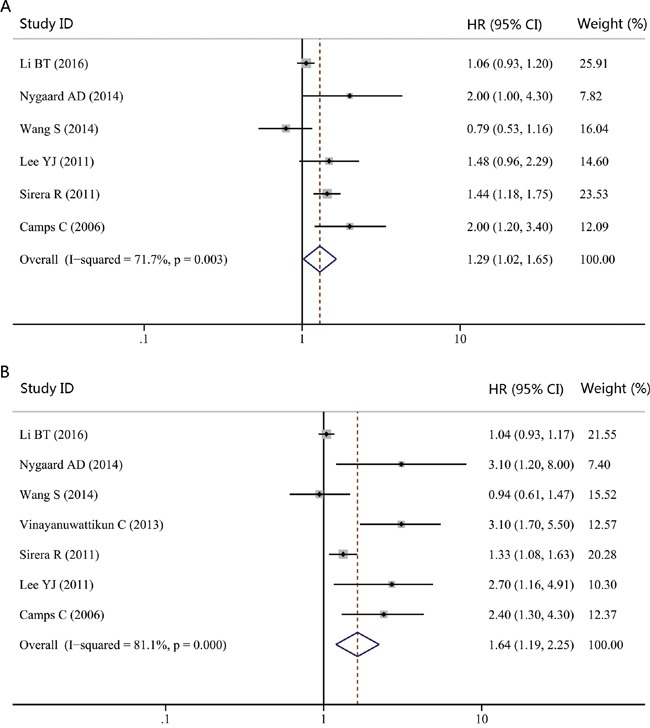
Forest plot of hazard ratio (HR) for the impact of cfDNA concentration on progression-free survival (PFS) and overall survival (OS) in NSCLC patients with advanced stages **A.** The impact of cfDNA concentration on PFS. **B.** The impact of cfDNA concentration on OS.

**Figure 5:**
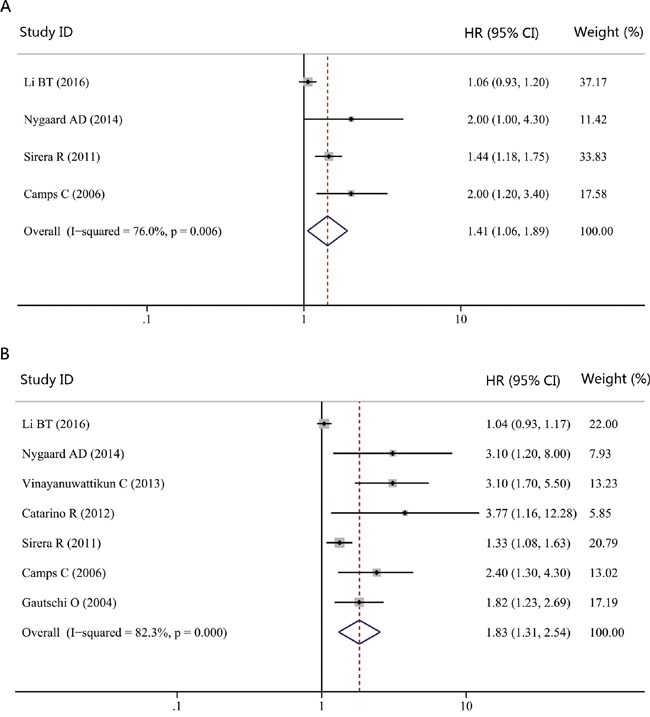
Forest plot of hazard ratio (HR) for the impact of cfDNA concentration on progression-free survival (PFS) and overall survival (OS) in NSCLC patients treated with chemotherapy **A.** The impact of cfDNA concentration on PFS. **B.** The impact of cfDNA concentration on OS.

As clinical stages and therapeutic regimens are correlated with patient's prognosis, they may bring heterogeneity to the overall analysis. Consequently, we focused on these two confounding variables in our subgroup analysis. As showed in Table 1, the majority of studies considered patients with advanced clinical stages (stage III-IV). Thus, we combined studies that focused on NSCLC patients with advanced stages to have a more homogenic group. The pooled HRs for PFS and OS were 1.29 (95% CI, 1.02-1.65; *P* = 0.035; *I^2^* = 71.7%; Figure 4A) and 1.64 (95% CI, 1.19-2.25; *P* = 0.002; *I^2^* = 81.1%; Figure 4B), respectively. We further performed another subgroup analysis according to the therapeutic regimens. As chemotherapy was the most commonly used treatment method in these studies, we then limited the analysis to studies considering patients treated with chemotherapy. The significant association could also be observed for both PFS (HR, 1.41; 95% CI, 1.06-1.89; *P* = 0.020; *I^2^* = 76.0%; Figure 5A) and OS (HR, 1.83; 95% CI, 1.31-2.54; *P* = 0.000; *I^2^* = 82.3%; Figure 5B).

### Impact of *KRAS* genotype detected in cfDNA on the survival of NSCLC

**Figure 6:**
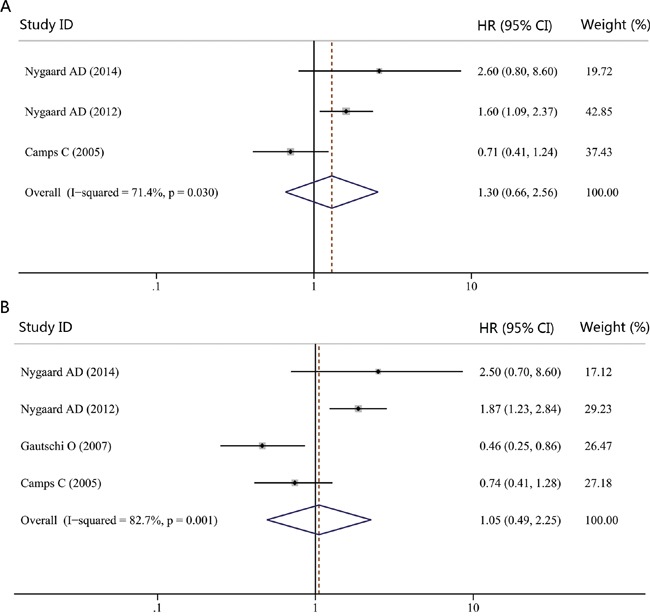
Forest plot of hazard ratio (HR) for the impact of *KRAS* genotype detected in cfDNA on progression-free survival (PFS) and overall survival (OS) **A.** The impact of *KRAS* genotype detected in cfDNA on PFS. **B.** The impact of *KRAS* genotype detected in cfDNA on OS.

The correlation between *KRAS* genotype detected in cfDNA with survival in NSCLC patients was evaluated in four studies. The combined HR for PFS was 1.30 (95% CI, 0.66-2.56; *P* = 0.450), suggesting that there were no significant differences between patients with *KRAS* mutation and those with wild-type genotype with respect to PFS (Figure 6A). Moreover, our study failed to reveal significant difference for OS when NSCLC patients were grouped according to *KRAS* genotype detected in cfDNA (HR, 1.05; 95% CI, 0.49-2.25; *P* = 0.892; Figure 6B). Thus, *KRAS* genotype detected in cfDNA might not be a prognostic factor for survival in NSCLC patients.

### Impact of *EGFR* genotype detected in cfDNA on response to EGFR-TKIs

**Figure 7:**
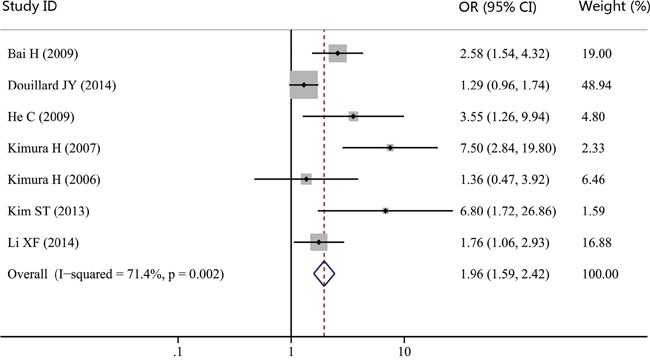
Forest plot of odds ratio (OR) for the impact of *EGFR* genotype detected in cfDNA on response to EGFR-TKIs

Seven studies evaluated whether *EGFR* genotype detected in cfDNA could act as a predictor of response to EGFR-TKIs. As showed in Figure 7, the pooled OR for objective response rates (ORR) was 1.96 (95% CI, 1.59-2.42; *P* = 0.000; *I^2^* = 71.4%). Our results suggested that patients with *EGFR* activating mutation in cfDNA had a greater chance of response to EGFR-TKIs. Thus, *EGFR* genotype detected in cfDNA may be a good predictor of response to EGFR-TKIs for NSCLC patients.

### Publication bias

**Figure 8:**
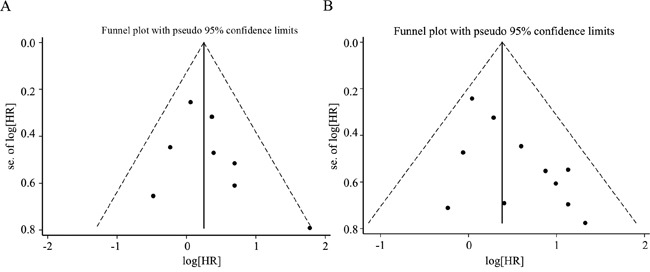
Funnel plot for the assessment of publication bias in this study **A.** Funnel plot for 8 studies reporting progression-free survival (PFS). **B.** Funnel plot for 11 studies reporting overall survival (OS).

We assessed the publication bias by visually assessing a funnel plot for asymmetry and by quantitatively performing Begg's test and Egger's test. As shown in Figure 8, there was no clear evidence of funnel plot asymmetry by visual assessment. Both Begg's test and Egger's test revealed that no publication bias was found when OS was analyzed (Begg's test, p = 0.266, Egger's test, p = 0.286). The Egger's test revealed a slight publication bias when PFS was analyzed (Begg's test, p = 0.119, Egger's test, p = 0.035). Thus, no significant publication bias existed in this study.

## DISCUSSION

Non-invasive approaches, usually based on plasma or serum samples, have showed great potential for treatment monitoring in NSCLC patients [47]. cfDNA, as an easily acquired liquid biomarker and a potential surrogate for the entire tumor genome, may provide complementary roles in predicting treatment response and survival of NSCLC patients. Many studies have investigated the usefulness of cfDNA as a screening tool for NSCLC. However, the predictive or prognostic role of cfDNA remains to be confirmed. In this study, we provided the evidence that high levels of cfDNA were significantly associated with poor survival in NSCLC. In addition, our study indicated that cfDNA could act as a promising predictive factor for response to EGFR-TKIs in NSCLC patients.

To the best of our knowledge, this is the first comprehensive meta-analysis to confirm the prognostic role of cfDNA concentration in NSCLC. Our study suggested that NSCLC patients with higher levels of cfDNA tend to have shorter PFS and OS time. One explanation for our results might be that total cfDNA was able to reflect the underlying tumor burden. Many studies had indicated that tumor cell lysate is the main source of the DNA found in plasma or serum [12]. Besides, the amount of cfDNA in the blood was significantly higher in NSCLC patients than that in healthy controls [9, 48]. What's more, cfDNA levels were associated with tumor volume, tumor stage, lymph node involvement and tumor responses [13]. Newman et al. found that levels of cfDNA significantly correlated with tumor volume and provided earlier response assessment than radiographic approaches [49]. Thus, patients with higher tumor load might have more intensive cfDNA released to the blood and cfDNA levels could reflect the tumor burden. On the other hand, cfDNA levels can be regulated by treatment-caused cell death. In NSCLC patients, an obvious transient rise in cfDNA concentrations occurred immediately after treatment and then it was followed by a rapid decrease [50]. It suggested that cell death caused by treatment could release cfDNA, which decreased as the tumor regressed. These observations revealed that cfDNA levels in plasma or serum were able to reflect the tumor load. Thus, cfDNA can be a surrogate for tumor burden, making it become a valuable prognostic factor for patients with NSCLC.

Targeted therapy based on molecular characterizations has greatly influenced the treatment strategies in NSCLC. Gene mutation analyses are the commonly used predictive biomarkers for selecting NSCLC patients to receive targeted agents. However, the current mutation analyses are often based on tumor tissues and have many limitations. First, the accessibility of tumor tissues is not always satisfactory as most NSCLC patients are diagnosed with advanced stages and unsuitable to provide tissues through invasive surgery or biopsy. Second, surgery and biopsy are not without clinical complications. The adverse events rate for thoracic biopsy was reported to be approximately 20% [51]. Furthermore, some percentages of NSCLC patients will develop resistance to molecular-targeted agents [52, 53]. Assessing treatment resistance in real time by repeated surgery or biopsy is not feasible. Considering these limitations, exploring convenient and less invasive techniques to monitor the therapeutic response and effects in NSCLC is urgently needed. Due to its nature of minimal invasiveness, cfDNA is a promising source for gene mutation analyses. In this study, we analyzed the impact of *KRAS* and *EGFR* genotype presented in cfDNA on the survival and response to EGFR-TKIs in NSCLC patients.

Approximately 15-25% of patients with NSCLC have *KRAS* mutations, resulting in constitutive activation of KRAS signaling pathways. Many studies reported that *KRAS* mutation could predict the poor outcomes of EGFR-TKIs treatment and chemotherapy, but several studies argued that *KRAS* mutation was not associated with the outcome of NSCLC patients [54]. A meta-analysis aimed to clarify the prognostic and predictive value of *KRAS* mutation in NSCLC was carried out recently [54]. Its results showed that *KRAS* mutation was significantly associated with worse OS and disease-free survival (DFS) in early stage NSCLC, and with inferior outcomes of EGFR-TKIs treatment and chemotherapy. However, statistical differences in DFS and PFS of chemotherapy and response rates to EGFR-TKIs or chemotherapy were not met when EGFR mutant patients were excluded. Our results indicated that *KRAS* mutations detected in cfDNA might not be a prognostic factor for the survival of NSCLC patients. One explanation might be that mutations of *KRAS* and *EGFR* were generally mutually exclusive in NSCLC [55, 56]. Most *EGFR* mutations were existed in *KRAS* wild-type patients, which might bias the results toward an overestimation of the prognostic and predictive value of *KRAS* mutation. Another reason might be that the amount of studies which assessed the prognostic value of *KRAS* mutation presented in cfDNA in NSCLC was small. Thus, the clinical significance of *KRAS* mutation detected in cfDNA is yet under debate. Future large-scaled trails are still needed to improve our results.

Nowadays, EGFR-TKIs are the most successful example of targeted therapy in NSCLC. *EGFR* gene mutations are the standard biomarkers for selecting NSCLC patients to receive EGFR-TKIs treatment. As a high degree of correlation between *EGFR* mutations detected in tumors and those presented in cfDNA has been confirmed by two recent meta-analyses [17, 18], *EGFR* mutation presented in cfDNA may also be useful predictive markers for guiding NSCLC patients to receive EGFR-TKIs. Indeed, several studies have analyzed the association between cfDNA *EGFR* mutation status and clinical outcomes. Goto et al. [44] found a significant correlation between cfDNA *EGFR* mutation status and PFS. In cfDNA *EGFR* activating mutation subgroup, NSCLC patients had longer PFS when they were treated with gefitinib. Another research demonstrated that *EGFR* mutation status in cfDNA was a good predictor for PFS after EGFR-TKIs treatment [57]. Consistently, our results showed that *EGFR* activating mutation in cfDNA indicated a greater chance of response to EGFR-TKIs in NSCLC patients. Thus, cfDNA *EGFR* mutation test had a good ability to predict the efficacy of EGFR-TKIs treatment. cfDNA might be a reliable material to guide EGFR-TKIs treatment for NSCLC patients.

However, there were some limitations in our present meta-analysis. Firstly, our analyses were based on the literature, making our results less reliable than individual patient data-based analysis. Secondly, a significant heterogeneity was presented in this study. When subgroup analyses were performed in terms of clinical stages and therapeutic regimens, the heterogeneity between studies did not change obviously. The heterogeneity might partly come from other variations, such as techniques that were adopted to detect cfDNA. Future standardization of cfDNA assessment would hopefully solve this problem. Thirdly, studies that could not provide sufficient data for extracting OR or HR were excluded. The exclusion of these studies might make the pooled estimates differ from their true value on some level.

In view of this study, our findings suggested that cfDNA could act as a predictive and prognostic biomarker for patients with NSCLC. High levels of cfDNA were significantly associated with poor PFS and OS in NSCLC. In addition, *EGFR* activating mutation status in cfDNA indicated a greater chance of response to EGFR-TKIs. In conclusion, cfDNA had a prognostic and predictive value for NSCLC patients, which might help to define high risk patients and guide clinical decision making. However, considering the limitations of a literature-based meta-analysis, these results need to be validated and updated by future large-scaled researches.

## MATERIALS AND METHODS

### Literature searches

Electronic searches for relevant articles in PubMed, Embase, and Web of Science databases were conducted in January 2016. The search strategy was generated by combining key words related to cfDNA (‘circulating cell-free DNA’ or ‘plasma cell-free DNA’ or ‘serum cell-free DNA’) and NSCLC (‘non small cell lung cancer’ or ‘NSCLC’). Moreover, we manually searched the reference lists of relevant articles for additional publications.

### Inclusion criteria

Studies were included in this meta-analysis if they met the following criteria: (1) all patients recruited in the study were diagnosed with NSCLC; (2) the predictive or prognostic value of cfDNA was evaluated; (3) only English-language studies were included; (4) the HR or OR and their corresponding 95% CIs were described or could be statistically extracted; (5) When several studies reported the same patient population, the newest or most informative study was included.

### Data extraction

Data extraction was performed independently by 2 reviewers and disagreements among them were resolved by consensus. The following information was extracted from each study: first author's name, publication year, country of origin, number of patients, therapeutic regimen, cfDNA assessment (methods), cfDNA analysis (quantification and molecular characterization) and clinical factors (PFS, OS and ORR).

### Statistical analysis

HR and its 95% CIs were used to estimate the prognostic value of cfDNA. OR and its 95% CIs were adopted to describe the correlation between cfDNA status and objective response rates. The individual HR or OR estimates were combined into an overall HR or OR, and the results were presented in the form of a forest plot. Pooled effect sizes were considered to be significantly different if their 95% CIs did not include 1 (p < 0.05). HR > 1 implied a poor survival and OR > 1 indicated a greater chance of objective response. Median pooled PFS and OS were summarized using descriptive statistics. The heterogeneity between studies was assessed by the Cochran Q test and *I^2^* test. When Cochran Q test P value was ≤ 0.10 and *I^2^* test *I^2^* value was ≥ 50%, statistically significant heterogeneity was considered to be present. Fixed effects models were employed when heterogeneity was absent; otherwise, random effects models were adopted. Funnel plots, Begg's test, and Egger's test were performed to detect publication bias. All analyses were carried out by using Stata Statistical Software, version 12.0 (Stata Corporation, College Station, TX, USA).
